# Fatal and nonfatal sharp force injuries to the limbs: a study of forensic autopsies in Sweden (2010–2019)

**DOI:** 10.1007/s00414-025-03554-7

**Published:** 2025-07-03

**Authors:** Anton F. Mittendorf, Nils Rosario Högberg, Tilde Joensuu, Yohan Robinson, Carl Johan Wingren, Ali-Reza Rezaie, Brita Zilg

**Affiliations:** 1 Swedish National Board of Forensic Medicine, Medicinaregatan 18C, Göteborg, 413 90 Sweden; 2 Department of Oncology-Pathology, Karolinska Institute, Stockholm, Sweden; 3 University of Gothenburg, Göteborg, Sweden; 4 Centre for Disaster Medicine, University of Gothenburg, Göteborg, Sweden; 5 Department of Forensic Medicine, University of Copenhagen, Copenhagen, Denmark

**Keywords:** Forensic medicine, Traumatology, Sharp force injury, Injury severity, Anatomical distribution, Injuries to the extremities, Abbreviated injury scale (AIS)

## Abstract

**Background:**

Sharp force injuries display varying patterns depending on the manner of death (homicide, suicide, accidental). The purpose of this study was to analyse forensic autopsy data to examine the characteristics of sharp force injuries on the extremities, to differentiate between different manners of death and to examine demographic differences.

**Methods:**

This retrospective cohort study utilized forensic autopsy reports from the Swedish Forensic Medicine Database. Cases were selected based on sharp force trauma as the cause of death. Injuries to the arms and legs were analysed for severity using a modified Abbreviated Injury Scale (AIS) to assess the trauma burden on the extremities and toxicological data was gathered. Statistical analyses were performed to explore differences in injury patterns and toxicological findings across manners of death as well as between sexes.

**Results:**

The study included 823 cases, categorized as 403 homicides, 365 suicides, 26 accidents, and 29 undetermined deaths. Suicides were more likely to involve lethal extremity injuries, particularly to the forearms and wrists. The injury severity was significantly higher in suicides than homicides when considering only cases with extremity injuries. Toxicology results showed higher intoxication rates in accidental deaths.

**Conclusions:**

This study highlights the utility of analysing the anatomical distribution and severity of injuries to the extremities in differentiating between homicides and suicides. Additionally, demographic and toxicological factors, such as age and substance use, provide valuable insights into the circumstances surrounding sharp force deaths.

**Supplementary Information:**

The online version contains supplementary material available at 10.1007/s00414-025-03554-7.

## Introduction

In the forensic assessment of sharp force trauma, several critical factors must be considered to determine the manner of death as either homicide, suicide, accident or illness [1, 2]. Sharp force injuries can range from minor surface wounds to fatal injuries [3, 4]. The number and severity of injuries inflicted during an attack can vary significantly, which directly impacts the likelihood of survival for the victim [3–5].

Previous studies have highlighted several relevant factors, including the number and severity of injuries [3, 6–12], specific wound characteristics such as type, location, and direction [3, 8, 12–31], as well as contextual elements like toxicology findings, geographical and seasonal conditions, clothing damage, and the object used [6, 7, 9, 14, 17, 19–21, 23, 26, 32–34]. Sociodemographic characteristics of the deceased, including age, sex and ethnicity/nationality should also be considered [3, 8, 9, 11, 19, 32, 35–37]. In cases of homicide, this extends to known sociodemographic data concerning the aggressor and the relation between aggressor and victim [6, 7, 9, 11, 18, 20, 24, 38, 39].

From a forensic perspective, even a single fatal stab wound can present complex challenges [40], as the physician must try to find the physiological process leading to the fatal outcome in each individual case and in many countries also make an assessment of the manner of death [41, 42]. Various parameters have been proposed for distinguishing suicidal, homicidal and accidental deaths [1, 8, 24, 43, 44], but while some basic principles are agreed upon, no definitive consensus exists [23, 42].

Sharp objects, particularly knives, are the most frequently used instruments in homicides across many countries [45]. Until 2018, knives were the most common homicide weapon in Sweden [46], although an increase in firearm-related deaths has since shifted this trend [47]. Despite this, sharp force injuries remain a significant cause of death in Sweden, with a notable 30% increase in knife-related fatalities between 2010 and 2022 [46].

Worldwide, the typical victim of a sharp force homicide is a younger male [7, 45, 46]. While gunshots have become the most common homicide method male victims in Sweden, sharp force is the most frequent homicide method among female victims [46] and while a higher proportion of knife-related deaths are seen among the young, the majority of victims are over 30 [47]. In cases of suicide, the victims are typically older males, often exceeding the average age of homicide victims [7, 8, 15, 48, 49].

In determining the manner of death in sharp force trauma, the forensic pathologist’s findings are often essential [1]. These include identifying signs such as hesitation marks [17, 21, 23, 25–27], defence wounds [12, 17, 21, 23, 25, 28–31], clothing damage [19–21, 26], and rib injuries [21, 24]. Several studies have examined the anatomical distribution of wounds [3, 13–21, 50]. This also includes injured structures such as blood vessels [16, 19, 21], in association with manner of death. However, the mere location of wounds is not the only important factor; as mentioned, the number and severity of injuries should also be taken into consideration. Traumatologists use various scoring systems, such as the Abbreviated Injury Scale (AIS) [51], to predict mortality based on injury severity [52]. AIS was originally designed as a standardised system for the categorisation of injury severity and injury type in traffic accidents. Over the years, the use expanded to include other injury mechanisms as well as modified and used to facilitate data processing and comparisons between trauma centres [53]. In short, every injury is assigned a code, where the numbers indicate injured body region, type of anatomical structure, specific anatomical structure, type and severity of injury [54].

However, these systems, designed for use in trauma medicine, can be difficult to apply in forensic settings. In addition, forensic pathologists may not have the same experience in the use of the scorings system as clinical traumatologists. Modifications such as the Summed Abbreviated Injury Scale (SAIS) [55] have been suggested for forensic use, with some success in evaluating injuries to specific anatomical regions [11].

Research focusing on specific body regions from a forensic standpoint is rare but provides insights into particular aspects, such as defence wounds [12, 17, 21, 23, 25, 28–31]or hesitation marks [17, 21, 23, 25–27], the offender-victim relationship [7, 9, 18, 24, 38, 39], and wound characteristics, such as orientation [14, 21] and direction [24]. Most studies, however, focus exclusively on homicides and suicides, often neglecting accidental and undetermined manners of death. While distinguishing between homicides and suicides is a common problem in forensic medicine, presenting data on accidents and in cases where the forensic pathologist was unable to come to a conclusion regarding the manner of death increases understanding of the whole spectrum of injuries, as well as the characteristics of those uncertain cases every forensic pathologist strives to minimise, and where further research into these characteristics can help in that endeavour.

While some research has explored the differentiation between homicidal and suicidal sharp force injuries to the arms [7, 19, 49], or have focused on either defence wounds [29, 31, 56] or hesitation marks [27] studying the distinction between defence wounds and hesitation marks is quite rare [25, 57, 58]. The overall severity of these injuries also remains under-examined from a forensic perspective. Severe injuries to the arms, particularly those involving vascular damage to the brachial, radial, or ulnar arteries, are relatively common but are rarely fatal in modern hospital settings [59]. However, in scenarios lacking advanced medical care, these injuries can have serious outcomes [60, 61].

The aim of this study is to characterise the number and severity of sharp force injuries on arms and legs in forensic autopsies across all manners of death, as well as the distribution of age, sex and toxicological findings. It will map the anatomical locations of these injuries, assess vascular involvement where applicable, and evaluate whether the localized Summed Abbreviated Injury Scale for the Extremities (ExSAIS) can be used to quantify injury severity. Additionally, it seeks to determine whether differences in injury severity exist between suicides and homicides.

## Materials and methods

This is a retrospective cohort study based on all forensic autopsy reports between 2010 and 2019 in Sweden noting sharp force injuries. Apart from the time frame, we included all cases with place of death in Sweden and cause of death sharp force trauma. Exclusion criteria were cause of death due to late onset complications from the sharp force trauma (such as infections), rather from the trauma itself.

The reports were obtained from a Swedish Forensic Medicine Database search using ICD10 codes (W25, W26, W45, X78, X99, Y28, Y35, S40, S41, S44, S45, S46, S48, S50, S51, S54, S55, S56, S58, S60, S61, S64, S65, S66, S68, S70, S71, S74, S75, S76, S78, S80, S81, S84, S85, S86, S88, E920, E956, E966, E986) and corresponding codes in ICD9 with supplementary text searches using words such as “sharp”, “knife”, “cut” and “scissors” was applied to the database and to the cause of death certificates.

**Fig. 1:**
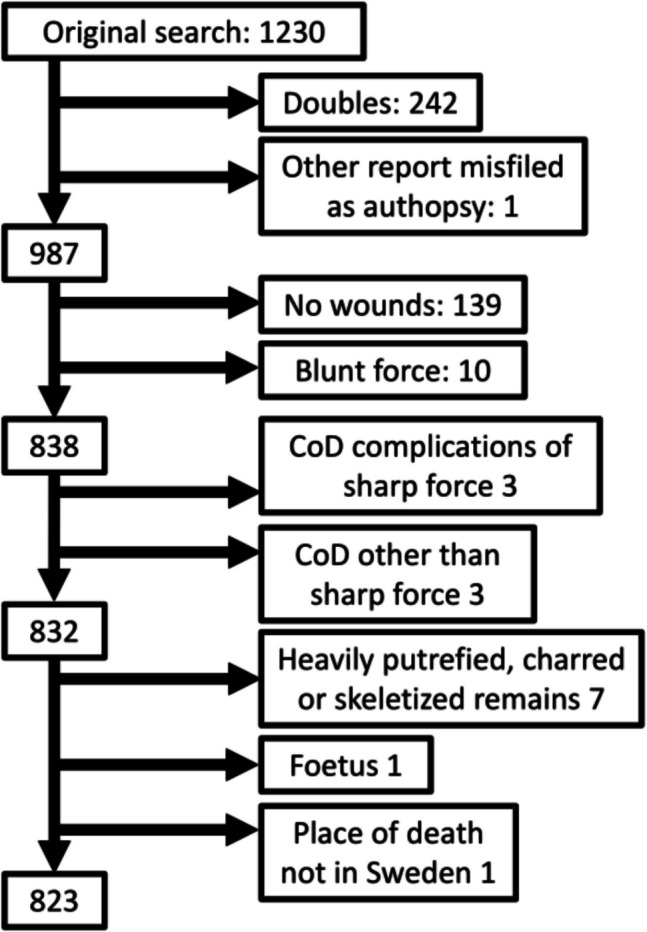
Exclusion steps

Cases where the main cause of death was other than the sharp force trauma (such as concurrent hanging) or secondary complications due to sharp force trauma (such as infections) were excluded, as were bodies whose condition made a proper forensic examination of the skin impossible (such as burnt bodies). For details, see Fig. 1.

The remaining 823 cases were categorized according to manner of death as (i) homicide, (ii) suicide, (iii) accidental or (iv) undetermined, as assessed by the forensic pathologist performing the autopsy. After two experienced forensic pathologists reviewing the autopsy reports and verdicts, the manner of death (as recorded in the database) was reclassified in the material in 10 cases (4 undetermined to homicide, 5 undetermined to suicide and 1 accident to suicide) in order to gain conformity of assessment between report and registry data. For more information see supplement X1. This resulted in 403 homicides, 365 suicides, 26 accidental deaths and 29 undetermined.

The accessory material, such as police reports and medical journals, was examined in the cases of suicide in order to assess the presence of background information agreeing with the case being a suicide.

All autopsy reports were examined to find the number (TNI, Total Number of Injuries), location and severity of the sharp force injuries on arms and legs, as well as any injured vessels and whether any of the injuries that had been judged as the cause of death, either by themselves of in combination with other wounds, were located on the arms or legs.

For the severity of each unique wound, the Abbreviated Injury Scale (AIS) 2005 was assigned each injury. The AIS assessment of injury severity ranges from 1 to 6, with 6 being an unsurvivable injury [62]. For details on how the AIS score was adapted to the current setting with addressing injuries on arms and legs see supplement X2.

When each wound had an AIS score, the sum of all these would yield the final ExSAIS score for each case, reflecting the trauma burden specifically for the extremities [10]. (E.g. six wounds with an AIS score of 1 each and two wounds with 3 each would both give an ExSAIS score of 6.) In order to compare TNI and ExSAIS, the “ExSAIS surplus” was calculated, subtracting TNI from ExSAIS. (As the base value of AIS is 1 per injury and vessel injuries generally are 3 or higher, an ExSAIS surplus of 2 in this case indicates an injury that is conceivably lethal by itself.) This makes it possible to examine whether there were more cases with a multitude of severe injuries in any category.

The anatomy of the arms was categorized as follows: (i) upper arm (including the elbow and the crook of the arm), (ii) forearm, (iii) wrist (defined as the area on or approximately within 3 cm proximally from the wrist flexion crease), and (iv) hand. The anatomy of the legs was categorized into: (i) upper leg (including the knee) and (ii) lower leg (including the foot). To avoid potential circular reasoning in the interpretation of specific findings, such as defence or handling injuries, we refrained from subdividing the forearm and wrist into more detailed regions (e.g., ulnar, volar, etc.), as such divisions might have influenced the assessment of the manner of death.

Forensic toxicology results were retrieved from a database maintained by the Department of Forensic Toxicology at the Swedish National Board of Forensic Medicine. Routine analysis was conducted on vitreous fluid, blood (preferably from the femoral vein), and urine, when available. To obtain the most accurate representation of the intoxication state at the time of death, we restricted the samples included in the analysis to blood and vitreous fluid. The toxicological findings were categorized into five groups: (i) ethanol, (ii) benzodiazepines, (iii) opioids, (iv) other sedatives, and (v) central stimulants. For the purpose of this study, the presence of toxic substances in categories (ii) through (v) was classified as either positive or negative, while ethanol was considered positive if the concentration exceeded 0.5 ‰.

## Statistical methods

Basic descriptive statistics was performed using Microsoft Excel 2019MSO, 64-bit version (Microsoft Corporation, 2019. *Microsoft Excel*; https://office.microsoft.com/excel). The anatomical location of injuries and injured vessels in categories of homicides and suicides were analysed using Mann–Whitney U test and Two Proportion Z-Test using R (version 4.3.1, R Core Team (2023). R: A Language and Environment for Statistical Computing. R Foundation for Statistical Computing, Vienna, Austria. < https://www.R-project.org/ >). We tested for sex differences using chi-square test using the online calculators on Social Science Statistics (Jeremy Stangroom 2024, used march 6th 2024; https://www.socscistatistics.com/) with a subsequent Benjamini–Hochberg adjustment using Carbocations Corporation’s online FDR Calculator (Carbocation Corporation, 2016, used july 19th, 2024; https://tools.carbocation.com). Odds ratios with logistic regression were calculated using R (version 4.3.1).

## Results

### Age, sex and toxicology

The majority of cases were young adults to middle aged men (Table 1). Lethal injuries to the extremities were more common among accidents and suicide victims compared to other manner of deaths (Table 2). Cases where at least one wound to an am or a leg was deemed the cause of death, either by itself or together with other injuries, had a different anatomical distribution than the overall number of cases with wounds on the extremities, with a far lower number of the homicides having a mortal extremity wound.

**Table 1:** Demographic data

Age group	Female	Male	All
0–19	10	35	45
20–39	58	204	262
40–59	60	219	279
60–79	50	142	192
80–99	9	36	45
	187	636	823

**Table 2:** Cases of lethal sharp force trauma according to manner of death (homicide/suicide/accident/undetermined), categorised by sex (male/female/all) and by presence of wounds on the extremities (any wound/mortal wound). Total number of cases in each category is presented without brackets, while percentages are within brackets. Percentages of cases with extremity wounds refer to the total number of cases in the category, whereas the percentages of cases with mortal wounds refer to the numbers of cases with extremity wounds, i.e. both refer to the number in the column one step to the left

	All			Male			Female		
Manner of death	Total	With extremity wounds	Of which mortal	Total	With extremity wounds	Of which mortal	Total	With extremity wounds	Of which mortal
Homicide	403	294 (73%)	40 (14%)	293	204 (67%)	37 (18%)	110	90 (82%)	3 (3%)
Suicide	365	268 (73%)	193 (72%)	295	210 (71%)	151 (72%)	70	58 (83%)	42 (72%)
Accident	26	26 (100%)	25 (96%)	20	20 (100%)	19 (95%)	6	6 (100%)	6 (100%)
Undet	29	20 (69%)	13 (65%)	28	19 (68%)	12 (63%)	1	1 (100%)	1 (100%)

**Fig. 2:**
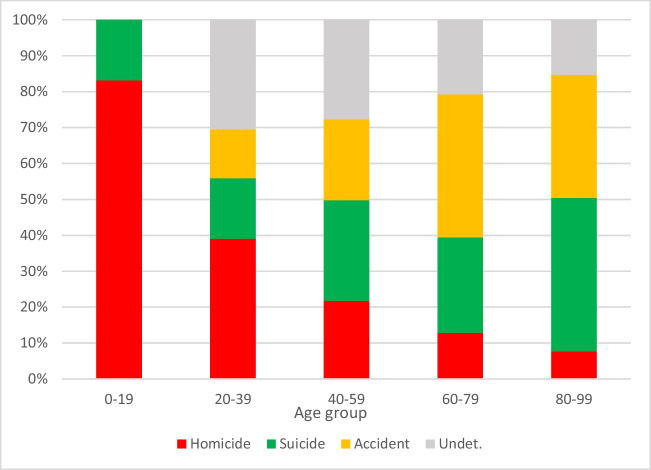
Percentages of different manners of death according to age group

**Fig. 3:**
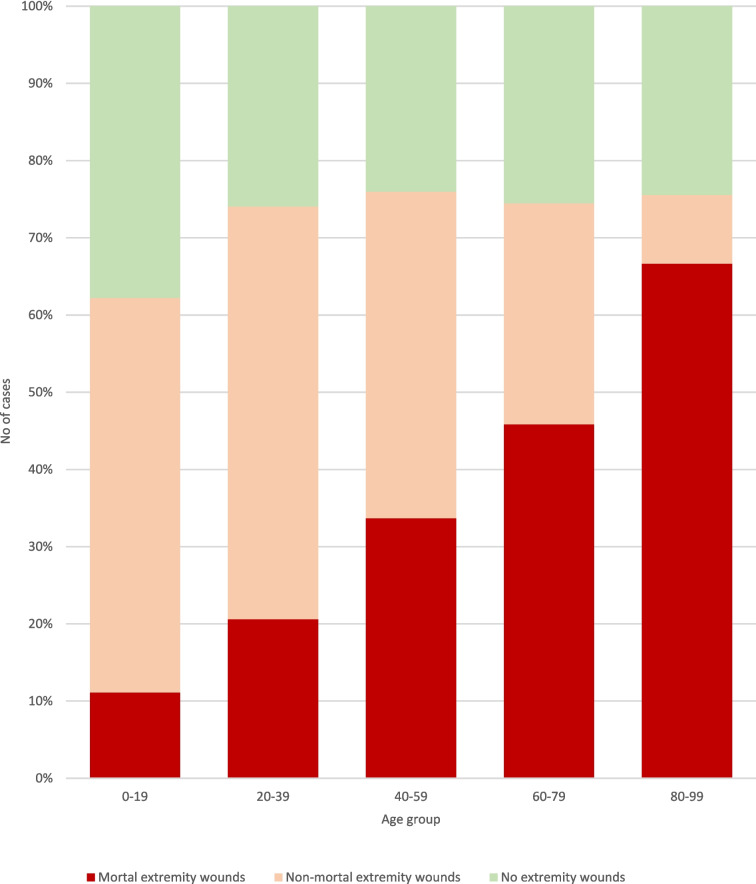
Numbers and distribution of cases with mortal wounds, non-mortal wounds and no wounds on the extremities, according to age group

When stratified into age brackets, an increasing ratio of suicide to homicides with increasing age was noted (Fig. 2). As could be expected from the higher percentage of fatal wounds to the extremities in suicide cases combined with the higher suicide incidence in the elderly population (Table 2 and Fig. 2), the percentage of cases with mortal wounds on the extremities rose with increasing age (Fig. 3). The percentage of sharp force deaths without any injuries to arms or legs appeared to be fairly constant, around 25%, apart from the youngest age bracket.

In the suicide cases, 84.9% of the cases (83.9% of men and 87.7% of women) had background material supporting the autopsy assessment of the manner of death.

The male population showed a larger proportion of suicides whereas homicide was more commonly seen in females (46.4% M to 37.4% F for suicides and 46.1% M to 58.8% F for homicides, adjusted p-value 0.024). The numbers when only including cases with wounds to the extremities were similar (46.4% M to 37.4% F for suicides and 45.0% M to 58.1% F for homicides, adjusted p-value 0.024), whereas the sex dominance was reversed in cases with mortal wounds to the extremities (68.9% M to 80.8% F for suicides and 16.9% M to 5.8% F for homicides, adjusted p-value 0.038).

In accidents, it was more common with intoxications of any kind (73% of cases) compared to homicides (52%) and suicides (49%). Alcohol was the most common finding (50% of accidents and 33% of homicides) with suicides being an exception in which only 12% tested positive. In contrast, benzodiazepines were more often identified in suicides (26%). (For figure, se supplement X3) The odds ratio (OR) calculations showed a significantly higher OR for intoxication among men, except for intoxications with benzodiazepines and central stimulants. There were also significant differences in OR between suicides/homicides, with the exception of opioids (Table 3).

**Table 3:** Numbers and percentages for different groups of toxicological findings (including data for any positive toxicological finding) in blood and vitreous fluid according to sex (Female/Male/Both sexes), odds ratio (OR), p-values and 95% confidence interval (CI) for toxicological findings according to sex, with male as reference and odds ratio, p-values and 95% confidence interval for toxicological findings (including the combination of alcohol and benzodiazepines) according to suicide/homicide, with suicide as reference

	Number (percentage) of findings			Sex odds ratio		Suicide/Homicide odds ratio		
	Female	Male	All	OR (95% CI)	*p*-value		OR (95% CI)	*p*-value
Alcohol	18 (10%)	187 (29%)	205 (25%)	0.23 (0.12–0.38)	< 0.001	**Alcohol**	2.69 (1.77–4.16)	< 0.001
Benzodiazepines	40 (21%)	130 (20%)	170 (21%)	1.29 (0.85–1.97)	0.241	**Benzodiazepines**	0.35 (0.22–0.56)	< 0.001
Central stimulants	17 (9%)	78 (12%)	95 (12%)	0.64 (0.34–1.12)	0.136	**Central stimulants**	3.40 (2.00–5.94)	< 0.001
Opioids	6 (3%)	41 (6%)	47 (6%)	0.39 (0.14–0.88)	0.037	**Opioids**	0.49 (0.24–0.96)	0.040
Other sedatives	7 (4%)	40 (6%)	47 (6%)	0.38 (0.14–0.87)	0.035	**Other sedatives**	0.16 (0.06–0.35)	< 0.001
Any finding	70 (37%)	352 (55%)	422 (51%)			Alcohol* **benzodiazepines**	5.69 (2.01–18.25)	0.002

The most common substances found (not including metabolites with their parent substances) were ethanol (*n* = 238), zopiclone (*n* = 55), amphetamine (*n* = 52), alprazolam (*n* = 43), nordazepam (*n* = 39), diazepam (*n* = 36) and dihydropropiomazine (*n* = 31).

### Anatomical distribution

**Fig. 4:**
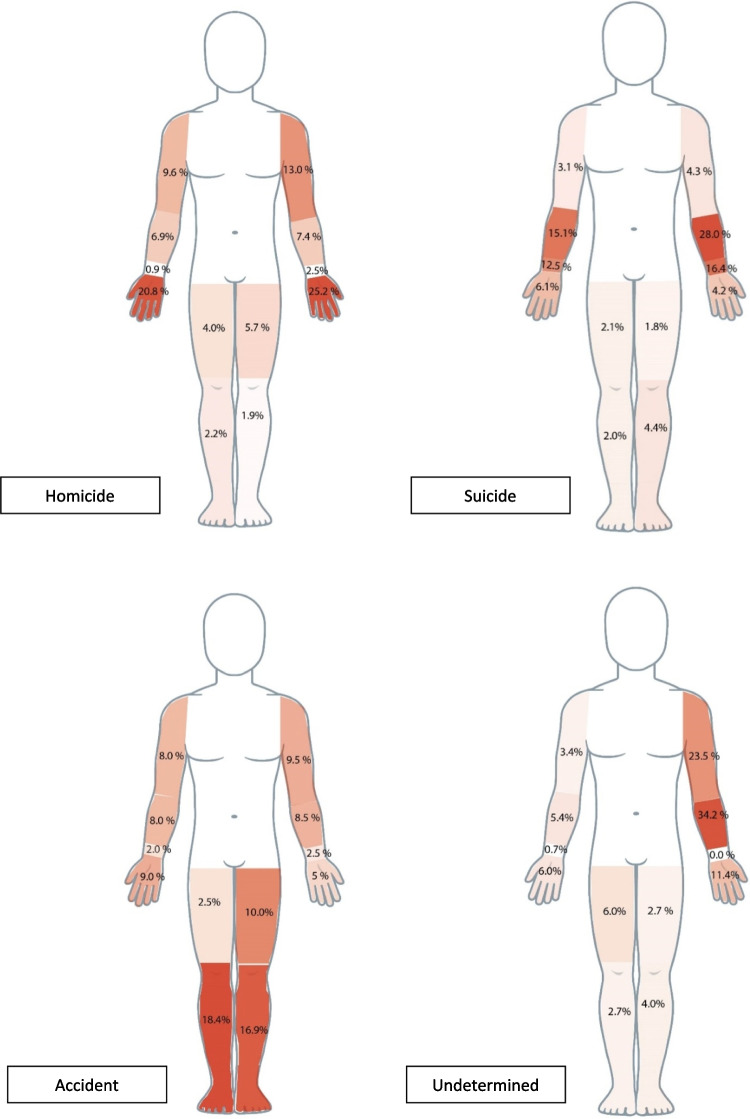
Anatomical distributions of wounds on the extremities according to manner of death

Seen as percentages, wounds on the forearms and wrists were more common in suicides, while homicides more frequently had injured hands and accidents more often injured legs (Fig. 4). For median number of wounds per body part, see supplement X4.

Other studies have shown that the majority of the deaths from penetrating trauma to the limbs result from injuries to the blood vessels [61]. In this study, the most commonly injured vessel was the radial artery in suicides, the large femoral vein in homicides and in undetermined cases, and the brachial artery in accidents. In 539 cases (364 homicides and 175 suicides) no injured vessel was described.

When comparing the total number of injured vessels reported in autopsies to those in cases involving fatal injuries to the limbs (Table 4), only minor differences in actual counts were observed. However, several homicide cases with fatal extremity injuries also presented non-fatal injuries to the upper arm veins. Significance testing using Two-Proportion Z-Tests, followed by Benjamini–Hochberg adjustments, revealed significant differences in the frequency of injured vessels. Specifically, significant differences were found for wrist arteries (*p* < 0.001 for both all cases and those with mortal wounds only), femoral/popliteal vessels (*p* < 0.001 for both), and upper arm arteries (*p* = 0.0034 and *p* = 0.036).

**Table 4:** Numbers and percentages on injured blood vessels, according to anatomical group, with separate calculation of cases with mortal injuries to the extremities. No = number, MOD = manner of death, w/= with, ves. = vessel, AV = arteriovenous

	All	Homicide				Suicide			
Injured vessel group	Total no of vessel injuries (all MOD)	No of vessel injuries	Of which mortal	% of homic. w/vessel injuries on limbs	Of which mortal	No of vessel injuries	Of which mortal	% of suicides w/vessel injuries on limbs	Of which mortal
Wrist arteries	120	7	7	17.9%	100%	109	108	57.4%	99.1%
Upper arm veins	33	5	2	12.8%	40%	29	28	15.3%	96.6%
Upper arm arteries	30	8	7	20.5%	87.5%	15	15	7.9%	100%
Femoral/popliteal ves	31	17	16	43.6%	94.1%	7	7	3.7%	100%
AV fistula	4	0	0	0.0%	N/A	2	2	1.1%	100%
Lower leg vessels	12	1	1	2.6%	100%	5	5	2.6%	100%
Forearm vessels	7	1	1	2.6%	100%	5	5	2.6%	100%
Neck and trunk ves	3	3	1	7.7%	33.3%	1	1	0.5%	100%
Unspecified vessel	30	2	1	5.1%	50%	31	28	16.3%	90.3%

### TNI/ExSAIS

The majority of observed injuries were minor (AIS 1), with mainly the larger injured vessels resulting in a higher score (AIS 2–4).

No significant difference could be seen when comparing ExSAIS or TNI for men and women. On average, homicide cases had the lowest ExSAIS surplus (ExSAIS minus TNI), indicating fewer severe injuries on average, whereas accidents were the only cases where the ExSAIS surplus was > 2 (Table 5).

**Table 5:** ExSAIS surplus (ExSAIS minus TNI) according to age groups and manner of death

Manner of death	Homicide	Suicide	Accident	Undet
All ages	**0.3**	**1.1**	**2.1**	**1.0**
0–19	0.1	0.9	-	-
20–39	0.3	0.9	1.5	0.4
40–59	0.3	1.2	2.1	1.4
60–79	0.3	1.2	2.2	1.6
80–99	0.7	1.4	2.5	0.0

**Fig. 5:**
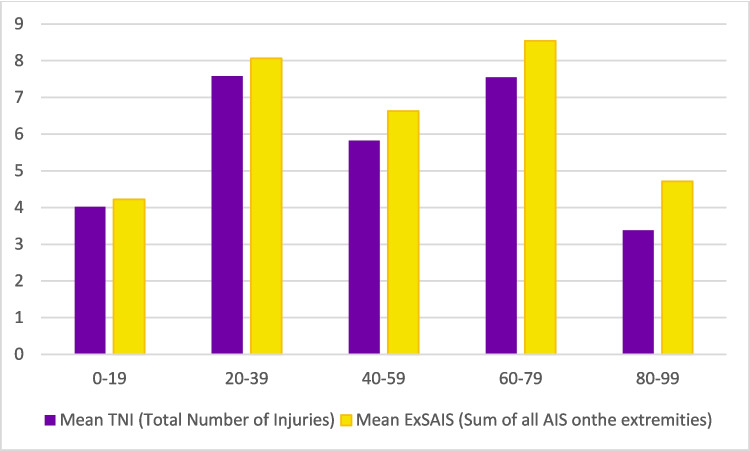
TNI and ExSAIS for different age groups

Overall, the mean number of wounds was lower in both the youngest and oldest age brackets. In most age groups, ExSAIS scores were slightly higher than TNI values, indicating greater severity per case. However, this pattern did not hold for the youngest and oldest individuals. In the youngest group, the mean injury severity was relatively low in relation to the number of injuries, suggesting less severe wounds overall. In contrast, the oldest group exhibited higher mean injury severity per wound on average (Fig. 5).

In all manners of death, the rise in injury severity to the limbs with increasing age could be seen, apart from the undetermined group, where only a single case over the age of eighty was present.

To evaluate whether ExSAIS is a more effective tool than TNI in distinguishing between homicide and suicide, both variables were tested for statistical significance using the full score for each case (i.e., the total number of injuries on both arms and legs combined). A Mann–Whitney U-test was conducted on the entire dataset, which did not reveal any significant differences. However, as ExSAIS is intended for use specifically in cases involving injuries to the extremities, a secondary analysis was performed, including only cases with at least one limb injury. This analysis yielded a significant difference for the ExSAIS variable, while the TNI variable remained non-significant (Table 6).

**Table 6:** Comparison of TNI and ExSAIS for homicides/suicides for all cases and for cases with at least one wound on the limbs, respectively, with Benjamini–Hochberg corrections

	*p*-value	adjusted
ExSAIS	0.099	-
TNI	0.801	-
ExSAIS, 1 + limb wounds	0.015	0.030
TNI, 1 + limb wounds	0.561	0.561

### Combined analysis

For the combined analysis, a multivariable logistic regression was performed to calculate the odds ratio (OR). Two models were developed: one that included cases with TNI values of 0 (i.e., no injuries to the extremities), and another that excluded these cases (Table 7).

**Table 7:** Regression analysis for multiple factors comparing suicide and homicide in all cases (left) and in cases with at least one wound to the extremities (right), with odds ratio (OR), p-values and 95% confidence interval, with suicide as reference

	All cases		Cases with at least one wound to the extremities	
	OR (95% CI)	*p*-value	OR (95% CI)	*p*-value
Female sex	1.87 (1.25–2.82)	0.003	1.58 (0.99–2.54)	0.056
Age	0.96 (0.95–0.97)	< 0.001	0.97 (0.95–0.98)	< 0.001
ExSAIS	0.50 (0.42–0.60)	< 0.001	0.46 (0.38–0.56)	< 0.001
TNI	2.00 (1.67–2.41)	< 0.001	2.17 (1.80–2.65)	< 0.001
Alcohol	1.95 (1.37–2.81)	< 0.001	1.57 (1.02–2.42)	0.041
Benzodiazepines	0.78 (0.50–1.20)	0.253	0.80 (0.48–1.34)	0.392
Central stimulants	2.21 (1.28–3.92)	0.005	2.30 (1.21–4.52)	0.013
Opioids	0.59 (0.28–1.20)	0.146	0.66 (0.29–1.50)	0.325
Other sedatives	0.17 (0.07–0.40)	< 0.001	0.18 (0.06–0.47)	< 0.001

The models were checked for interaction between ExSAIS and TNI. The test was non-significant and hence the potential interaction was excluded in the regression models.

For the total number of cases (i.e. including cases without injuries to the extremities) results were significant for all parameters except toxicological findings of benzodiazepines and opioids, but when limiting the analysis to only cases with wounds present on the limbs, the sex parameter ceased to be significant.

## Discussion

The objective nature of quantitative measurements facilitates cross-study comparisons and enhances the reproducibility of findings. However, in forensic research involving traumatic deaths, certain generalizations should be made with caution. Factors associated with homicide, such as hazardous alcohol and drug use [45], domestic violence [63], weapon accessability [64] and seasonal variations [9, 23, 36], have shown to differ significantly across countries. In contrast, based on the existing literature [65, 66], we found no evidence to suggest that other characteristics – such as the number, type, and anatomic al distribution of wounds – are influenced by cultural or national factors. These variables may therefore possess greater generalizability and hence may be better suited to a quantitative approach.

Nationwide Swedish data is particularly well-suited for such studies, as it includes all cases from the entire country. This minimizes potential bias caused by regional differences in socioeconomic status, immigration patterns, age distribution, urbanization, and other demographic variables.

The multivariable logistic regression shows that there are several characteristics that differentiate suicides from homicides, including age, sex, toxicological findings, total number of injuries (TNI) on the extremities and the combined severity of all injuries on the extremities (ExSAIS). We observed that homicides tend to have a higher number of injuries to the extremities on average, but that suicides have more severe injuries. The sex difference was only significant when including all cases, which may be due to the higher percentages of female cases with wounds to the extremities in general, as seen in Table 2.

While both ExSAIS and TNI showed significant results in the logistic regression, the SAIS system when analysed by itself reveals a significant distinction between homicides and suicides in relation to injuries sustained in the extremities (ExSAIS) when excluding cases with injuries confined to other body regions. This shows that the method is possible to use outside of logistic regression models. The finding reinforces SAIS validity as a classification tool in forensic trauma research and evaluation, giving researchers in forensic pathology a method of evaluating trauma severity numerically, without omitting the significance of minor wounds.

Previous studies have shown that the anatomical distribution of wounds are different in suicides and homicides [9, 14, 16, 19, 21, 48]. This study advances our understanding of the anatomical wound distribution by showing that examining the total number on injuries on different regions of the arms and legs still shows significant differences between homicides and suicides.

It is well known in the forensic medicine community that certain patterns of injury are more frequently seen in homicides and other patterns in suicides [25, 32, 36], especially the presence of defence wounds or hesitation marks on the arms. The characteristic locations and groupings of these particular injuries were not a part of this study but those injuries form, together with the location and severity of injuries, the framework for a forensic injury assessment on the extremities.

As demonstrated, injury severity can be used in conjunction with TNI to indicate differences between populations. This is exemplified by the age group analysis, which showed a clear trend of increasing overall injury severity with age, irrespective of the manner of death. The youngest age group was notable for a higher proportion of cases without injuries to the arms and legs. This may be attributable to the relatively lower number of suicides and accidental deaths in this group, as well as the increased vulnerability of younger individuals.

Sex-based differences were consistent with those reported in previous studies [8], although the proportion of males among suicide cases was higher in this study compared to some earlier reports [19, 23].

In comparison to Swedish data reported by Ormstad et al. [32], both male and female homicide in the present study were less frequently intoxicated with alcohol at the time of death (43% vs. 88% for males, and 9% vs. 30% for females). These findings more closely align with those of Gill and Catanese [23], who reported alcohol intoxication in 45% of male and 21% of female homicide victims.

The observed differences compared to earlier Swedish data may reflect changes over time in alcohol consumption patterns among younger Swedes, potentially influencing alcohol-related risk behaviour [67].

As this study relies on existing autopsy records, certain limitations concerning data quality are unavoidable. For instance, information on the specific blood vessel affected by the injury could not be determined in all cases based on the available documentation. In cases involving fatal injuries to the extremities, it is generally expected that the forensic pathologist will attempt to identify any damaged blood vessels. However, injured blood vessels were identified in only 77.5% of homicide cases with fatal extremity wounds, compared to 95.9% of similar suicide cases. This discrepancy is noteworthy given the critical importance of comprehensive forensic investigation in suspected homicides. One possible explanation is that, in certain instances, the extremity wound may have been regarded as part of a constellation of injuries collectively contributing to the cause of death, leading the pathologist to consider a detailed vascular assessment unnecessary.

In the Swedish system, the physician is responsible for determining the manner of death. This may introduce a risk of circular bias, as forensic pathologists are trained to recognize specific injury patterns, which could influence their interpretation in line with expected outcomes. To mitigate this risk, we employed an anatomical partition in this study that did not specify the anatomical aspects, which, particularly on the forearm, are traditionally associated with particular manners of death. Additionally, we assessed the extent to which suicide cases included non-autopsy information indicative of suicidal intent.

While the overall study population is fairly large for this type of study, some sub-categories were small in number, e.g. injuries to the neck and trunk vessels through injuries on the extremities. The findings regarding those sub-categories should therefore be interpreted with caution.

Another limitation is the result of the long time period investigated; as toxicological analysis methods have been refined and gradually implemented over time, the results from the end of the study period are not fully comparable to those from the start and the toxicology data should therefore be seen as indicators to be used together with the other data rather than a dataset to be interpreted on its own.

## Conclusions

There are many factors that differ when comparing manners of death from sharp force trauma, with particular importance placed on distinguishing between homicide and suicide. This study demonstrates that the anatomical distribution and severity of wounds, particularly in relation to injured blood vessels, are key variables that can support forensic assessments. While findings derived from mathematical analyses of injury patterns should not be applied directly to individual autopsies, they can help guide a more objective and structured approach to evaluating the manner of death as part of a broader decision-making process.

The identification and documentation of specific injured vessels in sharp force fatalities not only reflect differences in wound patterns between suicides, homicides, and accidents but may also deepen our understanding of the physiological processes leading to death. Although the percentage of cases with identified vessels was lower than ideal, our data suggest that reporting vascular injuries could improve both forensic interpretations and contribute to other fields. In particular, this knowledge may benefit emergency trauma care, where awareness of commonly affected vessels could aid in rapid intervention, as well as engineering and safety design, where understanding the lethality of certain injury types has practical implications.

Demographic and toxicological findings offer further insights. Notably, the oldest age group in this study exhibited the most severe extremity injuries, while the youngest cohort had the least, a pattern that held even when accounting for the manner of death. Suicides were more common in older individuals and were more frequently associated with fatal wounds to the limbs, while homicide victims tended to be younger and showed different injury profiles. Toxicological analysis revealed substance-specific patterns, with alcohol more prevalent in accidents and homicides, and benzodiazepines more common in suicides.

Taken together, these findings underscore the value of a multifaceted approach to sharp force fatalities, by combining injury pattern analysis, anatomical specificity, toxicology, and demographic context. Further studies are encouraged, particularly those exploring hesitation marks, defence wounds, and how forensic pathologists perceive and interpret injury patterns in practice.

## Supplementary Information

Below is the link to the electronic supplementary material.

Supplementary file1 (DOCX 14 KB)

Supplementary file2 (DOCX 15 KB)

Supplementary file3 (DOCX 19 KB)

Supplementary file4 (DOCX 19 KB)

## Data Availability

The data that support the findings of this study are available from the corresponding author, [AM], upon reasonable request.
