# Supplementary material for: Fatal and nonfatal sharp force injuries to the limbs: a study of forensic autopsies in Sweden (2010–2019)
Source: Int J Legal Med. 2025 Jul 3;139(6):2749–61. doi: 10.1007/s00414-025-03554-7 (PMC12532691; doi:10.1007/s00414-025-03554-7)
Supplement: Supplementary file 1 — Supplementary file1 (DOCX 14 KB) [file 414_2025_3554_MOESM1_ESM.docx]

**Supplement X1: Notes on reclassification of manner of death in orde to achieve conformity**

- Cases initially classified as undetermined due to unclear legal status reclassified as homicide after court verdict (2)
- Cases initially classified as undetermined in the cause of death certificate but noted as ”homicide” in the full report, reclassified as homicide (2)
- Cases initially classified as undetermined but specifically noted as self-inflicted in the autopsy report, reclassified as suicide (5)
- Complicated suicide, where the intention was to commit suicide, but the cause of death not the intended one, initially classified as accident, reclassified as suicide (1)
