# Supplementary material for: Fatal and nonfatal sharp force injuries to the limbs: a study of forensic autopsies in Sweden (2010–2019)
Source: Int J Legal Med. 2025 Jul 3;139(6):2749–61. doi: 10.1007/s00414-025-03554-7 (PMC12532691; doi:10.1007/s00414-025-03554-7)
Supplement: Supplementary file 2 — Supplementary file2 (DOCX 15 KB) [file 414_2025_3554_MOESM2_ESM.docx]

**Supplement X2: Notes on classification of injuries ccording to the AIS system**

- Wounds extending from an arm or a leg to another part of the body were included in the AIS classification, but injuries to anatomical structures in the other body part in connection to the wound were not. However, any injured vessel was noted for a separate analysis of injured vessels.
- If the wound extended to several extremities (such as both arms, crossing the torso) , all concerned extremities were classified as separate wounds.
- Injuries to nails were not counted.
- Injuries described as “several” of “multiple” were counted as n=3, but approximations (e.g.” around ten”) were counted as the given approximation.
- When irregularly shaped wounds were noted as being composed of overlapping wounds, the number of separate wounds given by the forensic pathologist was used.
- When separate wounds were deemed by the forensic pathologist as having been caused by a single attack, such as several finger injuries in line or entry/exit wounds, they were noted as n=1.
- Penetrating wounds from a screw driver were classified as sharp as were penetrating wounds from a dog bite, but penetrating wounds from a bull horn or severed limbs from machinery or tram wheels were not.
