# Supplementary material for: Fatal and nonfatal sharp force injuries to the limbs: a study of forensic autopsies in Sweden (2010–2019)
Source: Int J Legal Med. 2025 Jul 3;139(6):2749–61. doi: 10.1007/s00414-025-03554-7 (PMC12532691; doi:10.1007/s00414-025-03554-7)
Supplement: Supplementary file 3 — Supplementary file3 (DOCX 19 KB) [file 414_2025_3554_MOESM3_ESM.docx]

**Supplement X3**
