# Supplementary material for: Fatal and nonfatal sharp force injuries to the limbs: a study of forensic autopsies in Sweden (2010–2019)
Source: Int J Legal Med. 2025 Jul 3;139(6):2749–61. doi: 10.1007/s00414-025-03554-7 (PMC12532691; doi:10.1007/s00414-025-03554-7)
Supplement: Supplementary file 4 — Supplementary file4 (DOCX 19 KB) [file 414_2025_3554_MOESM4_ESM.docx]

Table X4: Median numbers of wounds (TNI) on each body region of the extremities, when present, according to manner of death.

|  | Manner of death | Left upper arm | Left fore-arm | Left wrist | Left hand | Right upper arm | Right fore-arm | Right wrist | Right hand | Left thigh | Left lower leg | Right thigh | Right lower leg |
| --- | --- | --- | --- | --- | --- | --- | --- | --- | --- | --- | --- | --- | --- |
| **All cases** | **Homicide** | 2 | 1 | 1 | 3 | 2 | 2 | 1 | 2 | 2 | 1 | 1 | 2 |
|  | **Suicide** | 1 | 3 | 2 | 1 | 1 | 3 | 2 | 2 | 2 | 2 | 1.5 | 3 |
|  | **Accident** | 3 | 2 | 1 | 2 | 2 | 1.5 | 1 | 1.5 | 3 | 2 | 1 | 2 |
|  | **Undet.** | 3.5 | 6 | 0 | 3 | 5 | 4 | 0 | 3 | 2 | 1 | 1 | 2 |
| **Cases with mortal wounds on arm and legs** | **Homicide** | 2 | 3 | 1 | 2 | 3 | 1.5 | 1 | 1 | 2 | 2 | 3 | 2 |
|  | **Suicide** | 1 | 3 | 2 | 2.5 | 1 | 3 | 2 | 2.5 | 2 | 3 | 1 | 3 |
|  | **Accident** | 3 | 2 | 1 | 2 | 2 | 1.5 | 1 | 1 | 4 | 2.5 | 1 | 2 |
|  | **Undetermined** | 4 | 6 | 0 | 7.5 | 5 | 4 | 1 | 3 | 2 | 2.5 | 1 | 1 |
